# Sedation and Anesthesia Options for Pediatric Patients in the Radiation Oncology Suite

**DOI:** 10.1155/2010/870921

**Published:** 2010-05-13

**Authors:** Eric A. Harris

**Affiliations:** Department of Anesthesiology, Perioperative Medicine, and Pain Management, Miller School of Medicine, University of Miami, 2574 Mayfair Lane, Weston, FL 33327-1506, USA

## Abstract

External beam radiation therapy (XRT) has become one of the cornerstones in the management of pediatric oncology cases. While the procedure itself is painless, the anxiety it causes may necessitate the provision of sedation or anesthesia for the patient. This review paper will briefly review the XRT procedure itself so that the anesthesia provider has an understanding of what is occurring during the simulation and treatment phases. We will then examine several currently used regimens for the provision of pediatric sedation in the XRT suite as well as a discussion of when and how general anesthesia should be performed if deemed necessary. Standards of care with respect to patient monitoring will be addressed. We will conclude with a survey of the developing field of radiation-based therapy administered outside of the XRT suite.

## 1. Introduction

Cancer continues to be a leading cause of pediatric mortality in the developed world, with physicians and scientists constantly developing new weapons to combat it. Chemotherapy, surgery, nutrition, and holistic medicine all have a place in the multimodal approach that can prolong longevity and ameliorate quality of life. As part of this armamentarium, external beam radiation therapy (XRT) has proven to be a safe and effective technique for the management of various malignant (and occasionally nonmalignant) lesions. XRT can be used for both curative and palliative purposes; in the latter case, children benefit from decreased pain, preserved organ function, and the maintenance of lumen patency in hollow organs [1]. The medical team, led by a radiation oncologist, often includes a physicist, a dosimetrist, several radiation therapists (technologists), the patient's primary care pediatrician, and often an anesthesiologist to direct the sedation and ensure patient safety [2].

Since radiation therapy is a painless procedure, many older patients can complete their treatment without the use of anesthesia or sedation. Parental reassurance, and possibly the promise of a small reward afterward, is enough motivation for many children to remain still. Clearly babies and younger toddlers are not receptive to such enticement, and these are the patients that make up the vast majority of XRT anesthesia cases. Older children may be distressed by the absence of a parent next to them, but they often respond well to pictures attached to the ceiling within their field of view or the presence of music in the room. Indicators that suggest the need for anesthesia include young age, anxiety, treatment complexity (e.g., prone position), emotional immaturity for age, and a history of noncompliance [3].

## 2. Alternate Site Anesthesiology

The provision of anesthesia for patients undergoing radiotherapy procedures may present a deceptively simple challenge to the anesthesiologist. These cases are often very short in duration, sometimes lasting no more than ten minutes, and can usually be accomplished without the use of general anesthesia. The patients are often healthy from a cardiopulmonary standpoint although some malignancies may be associated with other medical conditions (e.g., Trisomy 21) which increase the likelihood of cardiac anomalies, such as endocardial cushion defects. Furthermore, there is essentially no blood loss or fluid shift present. How then can we explain the discomfort that anesthesia providers experience when faced with performing cases in the radiation therapy suite? 

In general, many clinicians experience a palpable sense of angst when asked to do cases anywhere outside of the “*comfort zone*” of the operating room. The personnel employed in the XRT suite are well trained in their field of expertise; unfortunately for us, that field has little to do with anesthesiology. Assistance with lines, difficult airways, or anesthetic emergencies may be delayed or completely unavailable. Your colleagues and the anesthesia technicians might not be familiar with the location of the XRT suite, making it difficult and time-consuming to acquire personnel support, extra drugs, or equipment. However, the greatest source of concern seems to be the physical distance that must be maintained from the patients. While many alternate-site anesthetizing locations force the anesthesiologist to be at a considerable distance from the patient, perhaps even in a different room (CT scanner, MRI suite), the XRT area is unique in that there is no means of directly viewing the patient or the monitors. Instead, once the procedure has begun, we must rely solely upon the use of closed circuit television monitoring. While “*teleanesthsia*” has long been postulated as being a possible future direction of the field, few practitioners are excited about being the mavericks forced to incorporate this technology into their current practice.

## 3. Fundamentals of XRT

**Figure 1:**
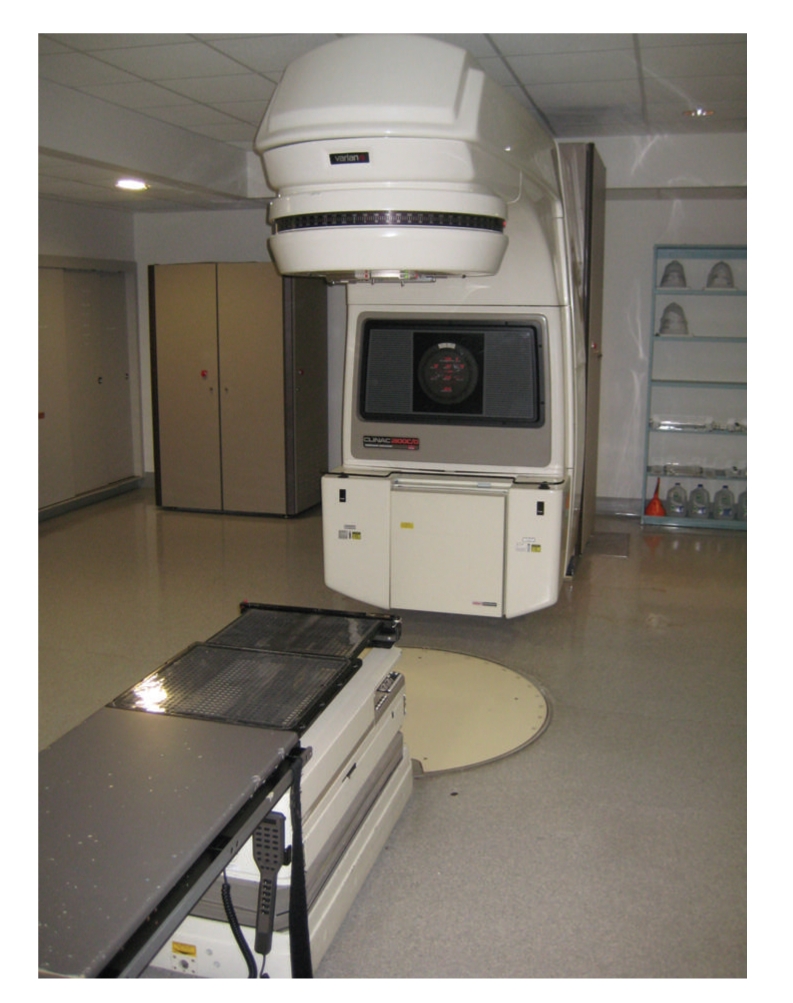
The linear accelerator.

Before anesthesiologists can feel more comfortable providing anesthesia in the XRT suite, they must first have a basic understanding of what is accomplished there. When the actual treatment room is first entered, the most obvious piece of equipment you will notice is the linear accelerator (Figure 1). Inside of this machine, electrons are accelerated to very high energy states within a vacuum. The electrons are then forced to collide with a material such as tungsten, which releases energy in the form of X-rays [4]. This energy is then focused at specific sites within the patient in an effort to degrade the genetic material within the tumor cells. The energy absorbed by the tissues is measured in terms of gray (Gy), which has replaced the more antiquated unit of rad. 1 Gy is equal to the deposition of 1 J/kg and is equivalent to 100 rad units [5]. While most patients receive this type of X-ray therapy, other types of lesions respond better to bombardment with electron, proton, or neutron beam therapy. In any event, the anesthetic considerations are essentially identical despite the type of subatomic particle that is utilized.

## 4. Simulation

**Figure 2:**
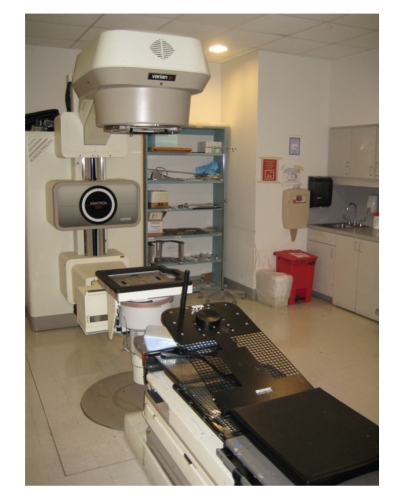
The simulation machine.

When a child is accepted as a candidate for XRT, he or she must first undergo a treatment planning session, referred to as a simulation. The physical set-up of the simulation suite is very similar to the XRT therapy room (Figure 2). However, the simulation machine is incapable of delivering therapeutic doses of radiation. Instead, it is used to provide radiographs of each treatment field which will aid the radiation therapy team in planning radiation doses and points of entry. Simulation serves several functions at the outset of therapy.

**Figure 3:**
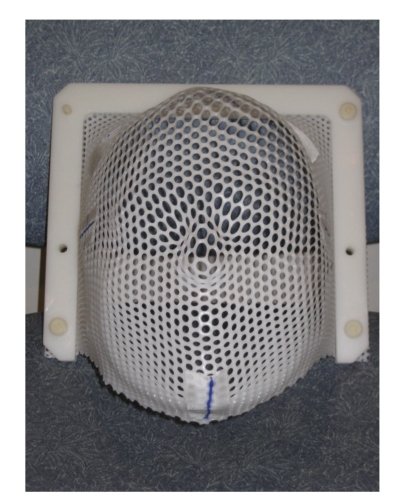
A premolded aquaplast.

**Figure 4:**
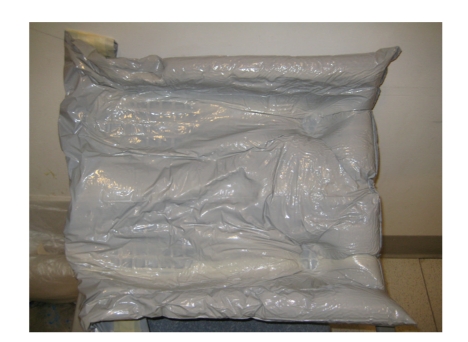
A premolded alpha cradle.

**Figure 5:**
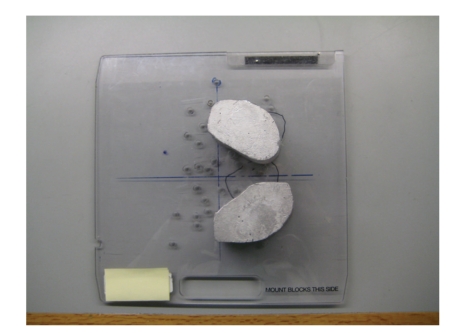
Blocks used to shield radiosensitive organs.

1. Simulation allows the radiation oncologist to prescribe the proper treatment by reproducing the exact conditions that will be encountered during the weeks of therapy. The number and location of anatomic fields that will need treatment will be decided; the radiation therapy team may typically treat anywhere from one to four fields, depending upon the type and size of the lesion. Ideal patient positioning will be also determined. Most patients can be treated in the supine position; however, craniospinal axis radiotherapy will necessitate the patient remaining in the prone position throughout therapy, while two lateral whole brain fields are supplemented with a posterior field of the spine [6]. This adds another layer of challenge to the anesthetic management.
2. Once these sites are determined, the therapist will mark the skin with ink to denote targets for future treatment. These markings will remain on the patient for the duration of the therapy and may be reapplied by the therapist as necessary. The markings increase accuracy and greatly enhance the speed of the future therapy sessions.
3. Plaster immobilization casts of the head (Aquaplast RT*™*, Q-Fix, Avondale PA, Figure 3) and/or body (Alpha Cradle, Smithers Medical Products Inc., North Canton OH, Figure 4) are made, depending upon the sites that are to be treated. These casts make certain that the child will not move during the treatment sessions, ensuring that the radiation is directed at its target and not at normal surrounding tissue. Inadequate immobilization can result in treatment failure [7, 8] as well as damage to normal tissue [9].
4. The radiation oncologist will determine if blocks will be necessary during the treatment period. Blocks are radio-opaque shields that are attached to the linear accelerator (Figure 5) to shield radiosensitive organs (e.g., kidneys and eyes) from the ionizing radiation.
5. If the team is still questioning the need for anesthesia, the simulation offers an ideal trial without the risk of radiation to see if the child will be cooperative and can remain immobile during the session.

The simulation session takes place anywhere from 20 to 90 minutes, depending upon the level of cooperation of the patient and the number and location of the fields that need to be marked. Most patients who will require anesthesia intervention for XRT will do well during the simulation with monitored anesthesia care (MAC). Since therapeutic radiation is not used, the anesthesia team can remain with the patient during the majority of the simulation. When conventional radiographs are taken, the anesthesia and radiation oncology teams can remain in the room while wearing lead shielding or safely observe the patient through a panel of leaded glass from an adjacent room. Medications can be given freely throughout the procedure as dictated by patient anxiety and motion. If general anesthesia is required, the anesthesia machine must be placed in a location that will not interfere with the lateral X-ray fields. Circuit hose extensions may be needed to place the machine at an appropriate distance from the patient. At the conclusion of the simulation patients may be recovered in the XRT suite, provided there is adequate nursing supervision. Alternatively, the patient can recover in the main postanesthesia care unit.

The simulation phase may immediately be followed by the first treatment, but it is more common for the family to return within the next day or two to begin the actual radiation therapy. This gives the team adequate time to map the coordinates of the sites that will be irradiated and decide upon dose and duration parameters. Total dose varies between 25 and 80 Gy, with a median value of 60 Gy. Lower doses are used for hematological cancers (leukemia and lymphoma) and seminomas; higher doses are reserved for solid tumors such as sarcomas and gliomas. The total dose of radiation is typically divided into 30 equal portions and administered once daily, five days per week over a 6-week period. Certain patients may benefit from hyperfractionated irradiation or the administration of XRT more than once daily [10]. Each field requires up to 90 seconds of irradiation; after this is completed, the radiation therapists must adjust the couch, reset the coordinates of the linear accelerator, and change the blocks so that the next field can be treated. Depending upon the number of fields (typically no more than four), the entire process can be completed in anywhere from 5 to 20 minutes. At specified time intervals (usually once per week), the therapists will repeat the radiographs to ensure that the anatomic targeting of the radiation beam is still accurate. This should add no more than another 5 minutes to the procedure.

## 5. Anesthetic Management of XRT Treatment

The majority of children who require anesthetic intervention can tolerate the daily therapeutic regimen with only MAC. Even patients who may have required general anesthesia for the simulation typically do well with moderate sedation (as defined by the ASA Task Force, Table 1) during the therapy phase, due to the brief time required for treatment. One significant exception is the child being treated for retinoblastoma; in this case, the globe must be kept completely immobile. MAC sedation, especially if ketamine is used (with a resultant lateral nystagmus), cannot accomplish this [11]. The room will be evacuated during the treatment period; however, it is safe to reenter in between doses, and therefore it is unusual to be away from the patient for more than 3 minutes. Of course, any reasonable request to reenter the room at any time should be honored by the radiation therapist; the treatment can be aborted before it is completed to allow safe entry into the room.

Parents are advised to follow fasting guidelines typical for all ambulatory surgical cases. If the tumor or medical condition is impairing gastric emptying, stricter guidelines may need to be enforced. Parents are encouraged to allow infants and children to ingest solid food and breast milk up to 4 hours before the procedure, and clear liquids are generally permitted up to 2 hours beforehand [13, 14]. Since fasting guidelines vary by institution, it is suggested that the practitioners follow the recommendations established by their own department.

**Figure 6:**
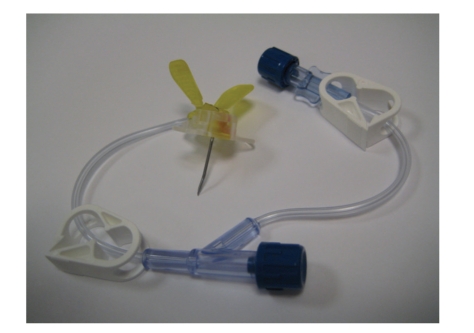
A Huber needle used to access an intravascular port.

The intravenous route is the preferred method of administering medication to these patients. While intramuscular drugs such as ketamine are effective, the repeated trauma of a painful injection daily for six weeks is often worse than the prospect of the XRT therapy. A large majority of these children have either recently completed a course of chemotherapy or are receiving it concomitant with the XRT and will therefore have an intravascular port present. Typically the port can be accessed with a Huber needle (Figure 6) prior to the first treatment, and the access can be left in place throughout the week and removed after the week's final treatment. The port remains dormant over the weekend, and the cycle repeats the following week. Parents can apply EMLA cream (AstraZeneca, London UK) to the site one hour before arriving Monday morning to make the access less traumatic. Alternatively, if the patient does not have a port, intravenous access via a peripheral vein can be obtained Monday morning, left in throughout the week, and removed on Friday, thereby following the same schedule [15]. Again, EMLA can greatly facilitate the process. In either case, the port or catheter should be flushed with a heparin flush solution (typically 300 units of heparin in 3 cc of normal saline) at the conclusion of each treatment to ensure continued patency throughout the week.

Aseptic technique is imperative when accessing a port or placing an intravenous catheter. These children are typically neutropenic from the XRT and/or chemotherapy, as well as their disease state, and cannot tolerate the threat of bacterial infection. Large case series estimate the risk of sepsis between 7% [3] and up to 15% [16]. The use of propofol, which can act as a potent culture medium for bacteria, may enhance the risk [17].

Fortunately, the advent of short-acting sedative agents has decreased the prevalence of such pediatric favorites as rectal methohexital [18], chloral hydrate, and the DPT cocktail (meperidine, promethazine, and chlorpromazine [19]). Intravenous midazolam has been the cornerstone of pediatric sedation since its introduction into clinical practice. The anxiolytic and amnestic profile is so good that many patients can complete their entire series of treatments with the aid of only this drug. If this is the case, participation of an anesthesiologist is rarely warranted [20]. The safety record of intravenous midazolam used in the absence of other sedative drugs is extensive. Since XRT is a painless procedure, it is unnecessary to supplement the benzodiazepine with narcotics. Therefore, with adequate monitoring of vital signs, the sedation can typically be managed by a registered nurse credentialed/trained in sedation, as per institutional protocol.

Patients who require more extensive therapy often still benefit from the use of midazolam. An initial dose of 0.05 mg/kg IV often provides enough sedation to allow for the placement of monitors. If ketamine is to be used, midazolam may decrease the incidence of postprocedure delirium [21]. After this initial dose of midazolam, the child should be dosed with a more potent agent to allow for transfer to the treatment couch and placement of therapeutic restraining devices. If necessary, midazolam can be readministered; cumulative doses greater than 0.2 mg/kg are rarely necessary. Of course, flumazenil must be readily available whenever benzodiazepines are being administered.

When benzodiazepine therapy is insufficient due to continued patient agitation, propofol is usually the preferred drug of choice for most anesthesiologists in the XRT suite, especially when dealing with children. After benzodiazepine pretreatment as previously described, an initial propofol bolus in the range of 0.5–0.8 mg/kg has been shown to provide adequate sedation for positioning and manipulation on the XRT couch while still allowing for spontaneous respiration and airway control [22]. This is followed by a continuous propofol infusion in the range from 7.4 mg/kg/hr [23] to 10 mg/kg/hr [24] throughout the treatment phase. Spontaneous eye opening was noted within 4 minutes of discontinuing the infusion [23]. Initial concerns about tachyphylaxis to propofol [25] have been disproved by more recent studies [26–28]. Thus propofol, combined with midazolam, provides excellent therapeutic conditions throughout the entire course of treatment [29]. Propofol has also been cited as being an excellent *stand-alone* drug to use in XRT without the need for benzodiazepine premedication. If the patient has a centrally accessed port, the likelihood of burning during propofol administration is highly unlikely.

The infusion of the *α*-2 agonist dexmedetomidine in the XRT suite has been described [30] although it has not been widely adopted. The most likely reasons for its infrequent use are the prolonged time needed to administer the initial bolus (which can be as long as the entire case itself), and the fact that pediatric administration of the drug constitutes an off-label usage.

Ketamine is another drug that is also used successfully, following midazolam pretreatment [31], to manage patients in the XRT suite [32, 33]. Ketamine can be given as a continuous infusion (25 mg/kg/hr) [34], but the *α*-phase serum half-life of 11 minutes [35] and the short duration of these cases often make this unnecessary. An initial dose of 0.5–0.75 mg/kg given at the start of therapy is often all that is required to accomplish the procedure. If the patient becomes agitated during the treatment, a supplemental dose of 0.25 mg/kg can be given to extend the period of cooperation. At some institutions, the use of ketamine has become so standardized that it is used in the XRT suite in the absence of an anesthesiologist [36].

Unlike propofol, it is not uncommon to witness tachyphylaxis develops to the effects of ketamine. By the fifth or sixth week of therapy the child may require twice the dosage to obtain the same effect as seen during the first or second week. Clinical experience has shown that recovery time is not prolonged in the latter phases of treatment, suggesting that the metabolism of the drug is also enhanced.

Fospropofol, a prodrug of the induction agent propofol, has recently been approved by the FDA for use as a sedative agent, to be administered by practitioners trained in the provision of anesthesia [37]. Like dexmedetomidine, its use in the pediatric population is currently considered an off-label usage. Fospropofol is converted in vivo by alkaline phosphatase to release propofol, formaldehyde, and phosphate [38]. Clinical studies suggest that an initial dose of 6.5 mg/kg, followed by a redose of 1.5–2 mg/kg if needed four minutes later, provides adequate sedation for minimally painful procedures with statistically insignificant incidence of side effects (desaturation below 92%, hypotension 20% below baseline [39, 40]). Burning on injection was not reported with fospropofol, but almost all patients report a tingling or burning sensation in the genital and perianal area [41, 42]. While fospropofol has not yet been widely marketed in the United States, it will be produced by the Esai Corporation under the trade name Lusedra [43]. Future clinical studies will determine its suitability in the XRT suite although its pharmacodynamic profile seems ideal for pediatric oncology cases.

When general anesthesia is required, the brevity of the procedure must be kept in mind when choosing an induction agent. A muscle relaxant may not be necessary (the exception, as stated before, is XRT for retinoblastoma, which requires paralysis of the extraocular muscles). The subglottic swelling that may develop with repeated daily intubations can be obviated by the use of a supraglottic airway such as the LMA (LMA North America Inc., San Diego, CA), [44, 45].

Antiemetic therapy is suggested at the conclusion of each day's treatment. The emetic effects of XRT can exacerbate the nausea from chemotherapy and stress and result in vomiting in the recovery area. Ondansetron 0.1 mg/kg is perhaps the agent of choice for most practitioners, but others report the use of steroids or phenothiazines with good results. Haloperidol, while showing some promise for the relief of postoperative nausea and vomiting, has been shown to be of little value in the XRT suite [46].

## 6. Nonpharmacological Methods of Anxiolysis

Some practitioners use psychosocial methods either in lieu of or as a supplement to pharmacologic sedation. These interventions may begin before the child enters the XRT suite. One center constructed an imitation linear accelerator in the children's playroom, complete with a large doll who received mock treatments. The children were allowed to act as the physicians and via transference were able to quell some of their apprehensions [47]. Other reports describe the use of music and videos [48], gradually immersing the patient by slowly introducing him/her to what is expected, rewarding each successful step [49], and using an interactive Barney character [50] in an attempt to keep patients calm and motionless. While the last study showed a statistically significant decrease in patients' heart rates, there was no difference in the incidence of observed behavioral distress or the need for sedation. Therefore, it is difficult to draw firm conclusions about the utility of these techniques. Furthermore, a busy XRT service might not be able to devote the necessary time and patience to foster the atmosphere necessary for such methods.

## 7. Monitoring during XRT

**Figure 7:**
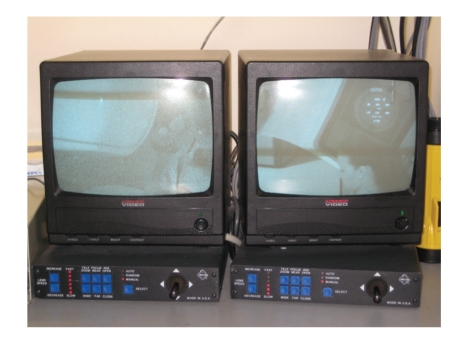
A remote monitor bank.

Remote monitoring of the patient receiving XRT therapy has progressed to the point where it is on par with technology found in the operating room. The days of rigging together makeshift monitoring devices [51] have been supplanted by the use of crystal clear closed circuit monitoring. The typical configuration (Figure 7) uses two cameras to provide visual monitoring. Each camera is controlled by switches next to the television screens, allowing individual control of zoom and focus [52]. One camera is directed at the patient to observe for consistent breathing and the absence of other movements. The other camera is focused upon the monitors, which typically include (at minimum) the ASA standards of EKG, NIBP, and pulse oximetry and qualitative end-tidal CO_2_. If the patient is receiving general anesthesia, the field of vision can be widened to include the ventilator and anesthesia machine as well. A microphone is also present to transmit the pulse oximeter tone. Remote audio monitoring of an esophageal stethoscope has been reported [53] but is not widely practiced. Documentation, either electronic or manual, should be completed from the initiation of sedation until the patient is transferred to a postanesthesia care provider.

## 8. XRT in Alternate Sites

The provision of radiation therapy is not limited solely to the XRT suite; indeed, it has begun to make inroads into the operating room. Brachytherapy or the intracavity implantation of radiotherapeutic material (e.g., radioactive prostate seeds and intrauterine isotopes) has been used successfully for years. Patient fears about “*becoming radioactive*” are largely exaggerated; because the radioactive material is sealed, only a small area around the site will be radioactive. The body as a whole will not emit radioactivity and it is generally safe for the patient to resume contact with others. In contrast, a patient receiving external beam XRT will emit no radioactivity whatsoever. Patients who receive intravenous radioactive isotopes, however, will continue to discharge radioactive material in their saliva, sweat, and urine. The duration of this is dependent upon the half-life of the agent used [54].

Surgeons, radiation oncologists, and anesthesiologists can also work as a team to provide intraoperative radiation therapy (IORT) [55]. This is especially useful for tumors which cannot be fully resected or have a high probability of local recurrence. In these cases, the treatment begins in the operating room, where surgical exposure and debulking of the tumor occurs. The wound is then covered, and the patient is then transported to the XRT suite to receive high-dose external beam radiation directly to the exposed tissue. The child is then returned to the operating room for closure of the surgical site. These cases, typically performed under general anesthesia, require a great degree of coordination between all parties involved. Transport of the patient with an open surgical site requires careful attention to maintaining a sterile field as well as continued provision of anesthesia and analgesia. The patient should be stable from a cardiovascular standpoint prior to leaving the operating room, and full monitoring, airway, and PALS supplies should accompany the patient during the transit phase [56].

**Figure 8:**
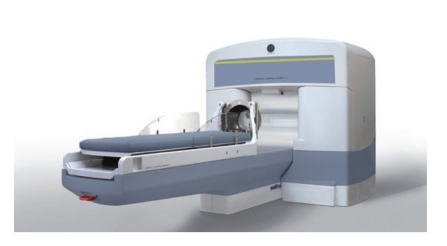
The gamma knife machine (courtesy of Elekta).

Stereotactic radiosurgery is another radiation therapy venue where anesthesiology services may be necessary. This procedure is used to treat conditions as diverse as malignancies, arteriovenous malformations, acoustic neuromas, and trigeminal neuralgia. The most widely used device, the Gamma Knife (Elekta Instruments Inc., Stockholm, Sweden), focuses 201 beams of gamma radiation (derived from cobalt-60) upon the lesion [57] (Figure 8). In contrast to XRT derived from a linear accelerator, only a single session of radiotherapy is needed to treat the disease. However, the patient may require several doses administered consecutively, each targeted to a different surface of the lesion.

Anesthetic management is much like what has been described for traditional XRT. MAC usually provides sufficient anesthesia although general may be required for very young patients and other special circumstances. The patient must first have the stereotactic frame placed which involves having four anchoring screws placed into the soft tissue of the head. The neurosurgeon or oncologist applying the frame will use local anesthesia to numb the areas; however, a small dose of ketamine or propofol immediately beforehand will make the procedure less traumatic. The child will then proceed to the MRI suite, where scans will be taken of the patient's brain with the external frame in place. It is imperative that all practitioners are aware of the hospital's protocols for MRI safety. The patient will likely be transferred to an MRI compatible stretcher, and all monitoring devices will be replaced with appropriate alternatives. Oxygen cylinders must be removed from the vicinity of the magnet. The patient's caregivers must be interrogated about the presence of any metallic implants, and the medical staff must remove any objects that may become a projectile hazard. Since the frame will limit access to the patient's airway, it is imperative that the patient is transported with the appropriate tools to quickly remove the frame in case airway access is necessary. If a vascular lesion is present, the child may also be taken to the neuroangiography suite for a diagnostic cerebral angiogram to further elucidate the anatomy. Afterward, the patient is permitted to rest while the physicians and physicists perform a 3D reconstruction of the MRI, plotting the coordinates that will most effectively target the intracranial pathology. The child is then placed into the Gamma Knife unit where several doses of radiation are administered (each lasting from 4 to 10 minutes). Upon completion, the stereotactic frame is removed, antibiotic ointment is applied to the puncture sites left by the screws, and the patient is transported to the recovery area.

The Cyberknife Robotic Radiosurgery System (Accuray Inc., Sunyvale CA) offers the clinical advantage of being able to treat tumors in any part of the body, freeing it from the intracranial restrictions of the Gamma Knife unit. Other enhancements include the Synchrony Respiratory Tracking System, a tracking software program that compensates for target movement caused by normal respiration. This obviates the need for a restrictive stereotactic device to be attached to the child, the primary reason anesthesia assistance is often requested for these patients. Thus, the absence of the frame and the freedom to relax and breathe normally mean older children can often tolerate this procedure with no pharmacological sedation.

## 9. Conclusion

Alternate-site anesthesiology has become more routine over the last decade as hospitals realize they can reduce costs and increase efficiency by “*outsourcing*” some types of cases out of the operating room. While some clinicians still feel uncomfortable emerging from the “protection” of the OR, others have embraced the chance to expand their practice beyond its traditional borders. XRT offers the anesthesiologist both a physical layout and a patient population that can be challenging initially but ultimately extremely rewarding.

## Figures and Tables

**Table 1:** Definitions of clinical states of sedation as proposed by the American Society of Anesthesiologist's task force on sedation and analgesia by nonanesthesiologists [12].

Sedation level	Characteristics
Minimal sedation/anxiolysis	A drug-induced state during which patients respond normally to verbal commands
	Cognitive function and coordination may be impaired
	Ventilatory and cardiovascular functions are unaffected

Moderate sedation/analgesia	A drug-induced depression of consciousness during which patients respond purposefully to verbal commands, either alone or accompanied by light tactile stimulation
	No interventions are required to maintain a patent airway, and spontaneous ventilation is adequate
	Cardiovascular function is usually maintained

Deep sedation/analgesia	A drug-induced depression of consciousness during which patients cannot be easily aroused but respond purposefully following repeated or painful stimulation
	Ability to independently maintain ventilatory function may be impaired
	Patients may require assistance in maintaining a patent airway, and spontaneous ventilation may be inadequate
	Cardiovascular function is usually maintained

	A drug-induced loss of consciousness during which patients are not arousable, even by painful stimulation
General anesthesia	Ability to independently maintain ventilatory function is often impaired
	Patients often require assistance in maintaining a patent airway and positive pressure ventilation may be required because of depressed spontaneous ventilation or drug-induced depression of neuromuscular function
	Cardiovascular function may be impaired
